# 3D Whole-Brain Imaging Approaches to Study Brain Tumors

**DOI:** 10.3390/cancers13081897

**Published:** 2021-04-15

**Authors:** Julian Taranda, Sevin Turcan

**Affiliations:** Neurology Clinic and National Center for Tumor Diseases, University Hospital Heidelberg, 69120 Heidelberg, Germany

**Keywords:** microscopy, brain tumors, volumetric imaging, in vivo two-photon microscopy, light-sheet fluorescence microscopy, serial two-photon tomography, neuro-oncology

## Abstract

**Simple Summary:**

Brain tumors integrate into the brain and consist of tumor cells with different molecular alterations. During brain tumor pathogenesis, a variety of cell types surround the tumors to either inhibit or promote tumor growth. These cells are collectively referred to as the tumor microenvironment. Three-dimensional and/or longitudinal visualization approaches are needed to understand the growth of these tumors in time and space. In this review, we present three imaging modalities that are suitable or that can be adapted to study the volumetric distribution of malignant or tumor-associated cells in the brain. In addition, we highlight the potential clinical utility of some of the microscopy approaches for brain tumors using exemplars from solid tumors.

**Abstract:**

Although our understanding of the two-dimensional state of brain tumors has greatly expanded, relatively little is known about their spatial structures. The interactions between tumor cells and the tumor microenvironment (TME) occur in a three-dimensional (3D) space. This volumetric distribution is important for elucidating tumor biology and predicting and monitoring response to therapy. While static 2D imaging modalities have been critical to our understanding of these tumors, studies using 3D imaging modalities are needed to understand how malignant cells co-opt the host brain. Here we summarize the preclinical utility of in vivo imaging using two-photon microscopy in brain tumors and present ex vivo approaches (light-sheet fluorescence microscopy and serial two-photon tomography) and highlight their current and potential utility in neuro-oncology using data from solid tumors or pathological brain as examples.

## 1. Introduction

As spatial molecular profiling technologies become accessible, efforts are focused on determining regional heterogeneity within tumors [1]. In parallel, the field of volumetric tissue imaging has gained significant momentum, and imaging technologies have been successfully applied to study oncogenic phenotypes in preclinical models and patient samples. As molecular and imaging technologies become more sophisticated, a synergy between these approaches is likely to emerge, paving the way for visual maps of molecularly annotated tumors. Ultimately, these integrated datasets have the potential to improve our understanding of tumors as a whole. This is an area where neuro-oncology has an advantage. The molecular brain tumor landscape has been studied extensively over the past decade, and the mouse brain has been the subject of deep imaging microscopy and associated analysis pipelines. Consequently, volumetric microscopy can be readily applied to the study of brain tumors to capture the detailed three-dimensional (3D) landscape of these tumors in the coming years.

**Figure 1:**
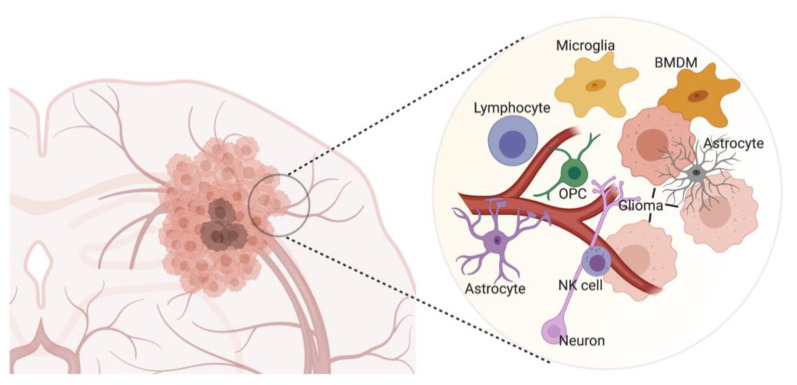
Overview of the glioma microenvironment. The brain TME includes microglia, bone marrow-derived macrophages (BMDM), astrocytes, oligodendrocyte precursors (OPC), neurons, lymphocytes, and natural killer (NK) cells. NK, natural killer cells; OPC, oligodendrocyte precursors; BMDM, bone marrow-derived macrophages. The figure was created with BioRender.com (accessed on 12 March 2021) and was exported under a paid subscription.

Brain tumors exhibit heterogeneity, integrate with the existing functional architecture of the normal brain, and reside within a complex 3D architecture of a tumor microenvironment (TME) (Figure 1). Thus, these tumors are not isolated entities but a disease of the entire brain. 3D imaging technologies allow us to visualize the dynamic behavior of these tumors in subregional volumetric space or create a static snapshot of the tumor throughout the brain. These modalities can shed light on many questions: How do tumor cells interact with each other? How do tumor cells affect the brain and vice versa? How do metastatic brain tumor cells infiltrate the brain parenchyma? To what extent do therapeutic agents cross the blood–brain barrier (BBB), and how do tumors respond to therapeutic intervention? 3D imaging modalities that map the interactions between brain tumors and their TME are likely to provide much-needed new insights into the interplay between the cellular structures of this complex ecosystem.

### Advantages of 3D Microscopy Approaches

Traditionally, two-dimensional (2D) methods relying on thin tissue sections, such as immunohistochemistry (IHC) and immunofluorescence, have been used to study the distribution of cells within tumors. These methods have several shortcomings. First, cross-sectional images do not fully represent tumor heterogeneity. 3D morphological structures such as tumor vasculature or neurons cannot be represented as continuous structures in 2D space. Second, reconstructing thin serial sections can provide high-resolution 3D maps [2], but such methods can be tedious and time-consuming. Third, cellular structures can be damaged by mechanical distortion during standard histopathological processing of thin sections [3]. Therefore, these methods are not ideal for volumetric reconstruction and quantification of large tissues such as whole organs and clinical specimens. This is an area where 3D imaging can complement 2D methods in several ways. Cells can be tracked longitudinally in their host environment, allowing the study of cellular dynamics in their natural habitat. In ex vivo whole-brain imaging, cell morphology and tissue structure remain undisturbed and can be visualized as a 3D projection. Furthermore, after ex vivo imaging, the tissue can be sectioned and used for molecular sequencing approaches or histology in follow-up studies.

**Figure 2:**
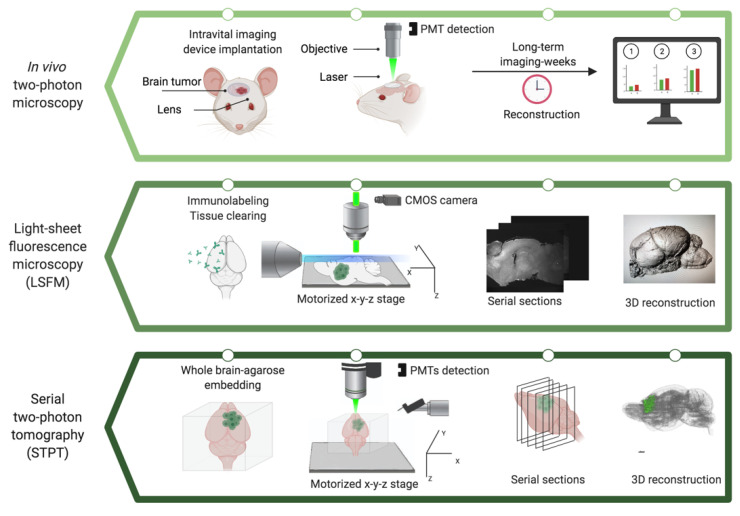
Schematic of 3D microscopes and their workflows for tumor reconstruction. Example reconstructed brains are shown for light-sheet fluorescence microscopy and serial two-photon tomography. PMT, photomultiplier tube; CMOS, complementary metal-oxide-semiconductor; 3D, three-dimensional. The figure was created with BioRender.com (accessed on 12 March 2021) and was exported under a paid subscription.

In recent years, there have been remarkable advances in both in vivo and ex vivo 3D imaging techniques. Here, we summarize the current state-of-the-art developments in high-resolution 3D imaging, focusing on in vivo (intravital imaging with two-photon microscopy) and ex vivo technologies (light-sheet fluorescence microscopy and serial two-photon tomography-(STPT)) and provide examples of their current or potential applications for brain tumor studies (Figure 2).

## 2. In Vivo Two-Photon Microscopy: Visualization of Longitudinal Biological Processes

Two-photon microscopy (2PM) is the most widely used form of multiphoton microscopy and was first demonstrated in 1990 [4]. Unlike confocal microscopy, which provides single-photon excitation, 2PM excites the fluorochrome by near-simultaneous (within <1 femtosecond of each other) absorption of two longer wavelength photons, which can be detected by the photomultiplier tube (PMT). Longer wavelengths scatter less and offer several advantages, including minimal photodamage to surrounding tissue, greater imaging depth (~1 mm), and reduced autofluorescence and photobleaching effects. Typically, an epicollection system is used in which all light collected by the objective is directed to the detector [5]. Therefore, in vivo 2PM enables high-resolution and dynamic imaging in live animals with cellular resolution (known as intravital microscopy, IVM) [6]. The first reported longitudinal two-photon IVM studies were performed in the field of neurobiology [7,8]. Since then, two-photon IVM has provided important insights into the dynamics of normal and pathological processes in the brains of living mice.

### 2.1. IVM Technology

The thickness and inhomogeneity of the intact mouse skull present a barrier to high-resolution optical imaging. Therefore, techniques such as a small craniotomy [9], thinning of the skull to a thickness of 20 μm [7,10,11], or chronic cranial windows of approximately 3 mm in diameter [8,12] have been developed. Thinned skull and cranial window approaches have their advantages and disadvantages; therefore, the choice of technique depends on the experimental design [13]. Cranial windows are implanted by replacing an area of the cranial bone with a glass coverslip to image the live brain [14,15,16,17]. Cranial windows in the cortex have proven useful in studying brain tumors [18]. In parallel, non-cortical window implantations have been developed to study brain tumors outside of the cortex. For example, cerebellar window implants have been used to study tumor-stroma interactions in preclinical models of medulloblastoma, a pediatric brain tumor that commonly forms in the cerebellum [19,20].

IVM studies are typically based on fluorescent proteins or exogenous fluorochromes (summarized in Table 1). These probes must be non-toxic, photostable, and sufficiently bright to produce a detectable signal through living tissue. The imaging depth of 2PM allows imaging of the subdural cortical layers in mice. Commercially available objectives typically support scanning with a high-resolution imaging field-of-view (FOV) of approximately 400 × 400 μm^2^ subregions [21], and more sophisticated mesoscopic arrangements can achieve a FOV of >9.5 mm^2^. However, these systems do not provide subcellular resolution in the axial dimension (>12 μm) [22,23,24]. Other two-photon mesoscopes can achieve a higher axial resolution (4.25 μm) with a FOV of 5 mm [24]. Although 3-photon microscopy and red-shifted fluorescence labeling can reduce the scattering effect, imaging depth in the mouse brain is still limited to 1 to 2 mm, and imaging quality decreases rapidly with increasing depth [25,26].

### 2.2. Application of Two-Photon IVM in Neuro-Oncology

Two-photon IVM (2P-IVM) is the most widely used approach for high-resolution 3D imaging in the preclinical brain tumor field, providing valuable insights into growth and invasion patterns [27,28,29], vascular dynamics [30], and immune cell infiltration [31,32] in the context of brain tumors and brain metastases. A previously unrecognized aspect of glioma biology was uncovered when 2P-IVM showed that brain tumor cells connect to form functional intracellular networks to transmit growth signals and resist conventional chemotherapy [33]. Therefore, pharmacological targeting of tumor microtubes may be a novel treatment modality for gliomas.

2P-IVM has been useful for characterizing the infiltration of metastatic tumor cells into the brain parenchyma. For example, in preclinical brain tumor metastasis models, circulating tumor cells have been shown to extravasate at vascular branch points, remain proximal to microvessels, and induce perivascular growth through vascular co-option or angiogenesis [34]. Tracking steps of brain metastasis revealed early activation of plasmatic coagulation by intravascular tumor cells, allowing colonization of the brain during metastasis [35]. A recent 2P-IVM study investigated the impact of the BBB on efficient drug delivery to the brain in preclinical models [36] and confirmed that increased BBB permeability is associated with increased tumor growth, and decreased permeability impedes effective treatment of brain metastases located behind an intact BBB. In this regard, IVM is likely to be an important tool for studies investigating the effect of therapeutic drugs on tumor cells and the accompanying TME. For example, the immune response to immunomodulatory drugs in preclinical immunocompetent brain tumor models can be measured using IVM technology.

An intriguing 2P-IVM study showed that intracranial biopsies could stimulate tumor associated microglia/macrophage (TAM) recruitment and trigger glioblastoma (GBM) progression [37]. This finding raises the question of whether an inflammatory microenvironment may contribute to tumor recurrence. Surgical resection remains one of the most commonly used but least studied components of glioma therapy, and gliomas invariably recur at the resection margin [38,39,40]. Therefore, there is a need to establish clinically relevant models of tumor resection and tumor recurrence. To address these challenges, IVM studies focusing on residual tumor cell growth patterns and the effect of TME in preclinical glioma resection models can be very informative.

One of the barriers to drug delivery to the brain tumor is the BBB. From this perspective, IVM can be a unique and important tool to study drug delivery to the brain. Drug analogs can be fluorescently labeled to allow pharmacokinetic measurements to determine the final destination of specific drugs along with their therapeutic effects on the tumor. Several conjugated drugs are available, such as the PARP-1 inhibitor AZD2281 modified to incorporate the fluorochrome BODIPY [41] or ibrutinib modified to include the fluorochrome SiR-COOH [42]. These studies would provide important insights into BBB permeability and dose-response relationships. Ultimately, these parameters can be integrated into an interactive feedback loop for rational design and optimization of therapeutic drug delivery.

Microglia constitute mostly of immune cells in the brain TME, and it has become clear that TAMs actively influence brain tumor biology [43]. Marker similarities between microglia and other myeloid cells pose a challenge to distinguish between the different subtypes of the central nervous system (CNS)-resident microglia (microglia, border-associated macrophages in the meninges, choroid plexus, and perivascular spaces) and circulating myeloid cells (BMDMs) that infiltrate the CNS during glioma pathology. In recent years, several experimental tools have been developed, such as antibodies targeting microglia or reporter mice to track microglia populations, such as the *Cx3cr1^GFP^* mice [44]. IVM can be used to map and differentiate the responses of brain-resident microglia and BMDMs to tumor formation and therapeutic interventions such as CSFR1 inhibitors that target TAM populations. For example, 2P-IVM has shown that infiltrating and resident myeloid cells have unique compositions, different migratory capacities and functions in in GBM [45]. As the toolkit for labeling microglia subpopulations becomes more granular, IVM is poised to continue to make important contributions to the dynamic roles of TAMs in glioma biology.

Moreover, 2P-IVM can be correlated with various approaches such as MRI or focus ion beam/scanning electron microscope (FIB/SEM) to obtain multilayered and informative datasets. Iron oxide nanoparticles are preferentially phagocytosed by TAMs can be detected by MRI to monitor immune responses [46]. Using this strategy, a recent study coupled fluorescently labeled iron oxide nanoparticles with 2P-IVM to longitudinally track the innate immune response by replacing the conventional titanium rings with Teflon cranial windows to reduce susceptibility artifacts in MRI [47]. In addition, 2P-IVM can be combined with FIB/SEM techniques to obtain ultrastructural images of selected regions of interest [48].

### 2.3. In Vivo Two-Photon Microscopy: Challenges

The brain was the first organ to be imaged in vivo using 2PM, and methods have since been developed to improve image quality. While in vivo 2PM has shed unprecedented light on pathological processes associated with brain tumors, some limitations of the technique should be considered. Most importantly, deep tissues such as the brain can only be imaged at reduced depth [49]. Improvements can be achieved by developing techniques that allow deeper photon penetration or provide deeper access to the tissue (e.g., via stick objectives or microendoscopes). Viewing the brain with endomicroscopy requires a small incision to implant the probe, and methods are being developed to minimize the damage and inflammation associated with the implantation [50,51]. For example, intravital two-photon endomicroscopy has been used to study glioma progression in the brain [52] and neuronal plasticity in the hippocampus [53]. These setups are of particular interest for studying brain tumors that originate in non-cortical areas such as the medulla (e.g., medulloblastoma) or brainstem (e.g., diffuse pontine glioma).

In addition, injury from optical imaging setups (e.g., cranial windows) is accompanied by inflammatory responses that may impact studies investigating vascular or immune system involvement in brain tumor pathology [54]. Thinned cranial windows may injure the pia if re-thinning is required. Furthermore, cranial windows for prolonged in vivo imaging produce an inflammatory response that can take up to two weeks to subside and allow physiological imaging [55]. This injury-induced inflammation can alter dendritic spine plasticity and lead to significant glial activation during the first month after surgery [56]. Long-term imaging with glass windows can also lead to the formation of granulation tissue, which can adversely affect the optical quality and fluorescence signal [57]. In addition, most 2P-IVM studies measure, at most, three parameters, which limits the number of cell types or structures that can be studied. However, studies have begun to address this challenge by developing a multiphoton imaging protocol to characterize the interactions between multiple cellular components in the living mouse [29].

To address some of the challenges described above, methods are currently being developed to improve temporal resolution and reduce photodamage using adaptive excitation [58], increase tissue penetration through the use of adaptive optics [59] or use three-photon microscopy [60], as well as long-term imaging techniques. Having already established itself as a useful tool, in vivo multiphoton microscopy will undoubtedly continue to contribute to discoveries in brain tumor pathogenesis.

## 3. Whole-Brain Volumetric Microscopy for Ex Vivo Imaging

Tumors or metastases can arise from a few malignant cells, and these single foci are difficult to detect with imaging systems limited by spatial resolution. Simultaneous visualization of multiple cell types or molecular markers will facilitate our understanding of the underlying molecular mechanisms and cellular co-options during tumor progression. In this context, the application of whole-brain imaging techniques to brain tumors may reveal interesting aspects of tumor biology. Robust whole-brain imaging techniques, software tools for volume rendering and 3D data analysis, and anatomical reference atlases for the mouse brain have been developed. These methods have successfully revealed various brain structures and functions in rodents, such as mesoscale projection patterns, single-cell-level neuronal tracing, and neuronal activity patterns [61]. These approaches provide a blueprint for understanding the basic brain structure and pathophysiology of brain disorders. In this review, we present two light microscopy-based approaches for automated 3D imaging of the whole brain based on either light-sheet fluorescence microscopy of chemically cleared tissue or the integration of automated block-face imaging methods with tissue sectioning.

### 3.1. Light-Sheet Fluorescence Microscopy

In the simplest form of a light-sheet microscope, the illumination light is formed into a sheet, and the fluorescence is detected with a camera perpendicular to the illumination axis. By moving the tissue sample vertically through the planar laser beam on a motorized *x-y-z* stage, a *z* stack of serial optical sections through the volume is obtained (Figure 2). In light-sheet fluorescence microscopy (LSFM), the thickness of the light-sheet can be adjusted to obtain different resolution in *z*, such that the thinner light-sheet results in a better *z* resolution. The focal plane is coplanar with the illumination sheet, and the sample is excited to collect the fluorescence signal. The setting of the light-sheet shape parameters allows that no out-of-focus regions are exposed to light and captures images of the entire section simultaneously depending on the size of the FOV and the magnification. In addition, this feature provides fast image acquisition limited only by camera performance. This design facilitates rapid 3D imaging simply by moving the sample using the motorized stage focal plane through the specimen to obtain serial section imaging depending on the setting. Compared to wide-field fluorescence, confocal, or multiphoton microscopy, LSFM is a robust technique with only the in-focus plane illuminated, minimizing photobleaching and phototoxicity.

The initial LSFM setup, known as selective-plane illumination microscopy (SPIM), was used for in vivo imaging of transgenic GFP-expressing embryos of medaka [62] and zebrafish [63]. In recent years, LSFM has been used for 3D microscopy of larger cleared samples. Earlier LSFM architectures such as ultramicroscopy [64] and CLARITY-optimized light-sheet microscopy (COLM) [65] have been used to image entire mouse brains within a few hours by placing the samples in a chamber filled with immersion medium with an orthogonally placed objective. The disadvantage of this geometry is that the samples are constrained by the chamber and the objectives, and exhibit progressively poorer image quality towards the center.

Other LSFM configurations that allow for laterally unconstrained imaging include dual inverted selective-plane illumination microscopy (diSPIM) and light-sheet theta microscopy (LSTM), which allow imaging of the entire postmortem human brain using thick sections of cleared human brain tissue (~10.5 mm × 14.1 mm × 3 mm) [66]. Although, both approaches have performance trade-offs in image quality and ease of setup. To improve the ease-of-use and throughput of LSFM, open-top light-sheet (OTLS) microscopes have been developed to overcome some of the limitations in sample size and shape for high-throughput imaging of large or multiple samples with micrometer-scale resolution, although this setup limits high-resolution imaging of small tissue volumes [67]. Other imaging modalities such as oblique light-sheet tomography (OLST) allow for high-resolution and uniform imaging of the entire mouse brain within ~14 h. In OLST, the illumination/detection is oblique to the sample, and the imaged upper portion of the tissue is removed by vibratome sectioning, similar to STPT (described in the next section) [68]. Recently, a new updated microscope version by LaVision called Ultramicroscope Blaze has been developed with improvements in the chamber capacity to allow the placement of multiple samples at once (multiple organs of a single mouse), and imaging sequentially with automated switching between different objectives and high-magnification lenses such as 12× with long working distance (16 mm) [69].

In general, LSFM images chemically cleared transparent tissues with a high resolution and short acquisition time, using only optical sections without mechanical sectioning. Subsequently, the acquired data can be reconstructed for 3D visualization of whole organs or tissues with micrometer resolution in the *x-y-z* axis. An important consideration for LSFM is optimized tissue clearing methods that render large samples highly transparent to improve light penetration, imaging depth, and contrast.

The concept of organic solvent-based tissue clearing was established in 1914 by Werner Spalteholz [70]. Initially, this hydrophobic clearing method followed various intensive steps that damaged the few centimeters of tissue on the surface [71]. Since then, various tissue clearing protocols have been developed, including three main approaches: organic solvent-based methods [64,72,73], hydrophilic chemical-based methods [74,75,76], and hydrogel-based methods [77]. All clearing methods attempt to minimize light scattering caused by mismatches between the refractive index (RI) of the tissue, which can range from 1.33 (water) to 1.600–1.604 for hydroxyapatite extracted from bone tissue [78,79], and the imaging solution. Tissue clearing may have the added benefit of eluting heme chromophore from hemoglobin, increasing tissue transparency to enhance image resolution [80]. Overall, effective tissue clearing methods aim to preserve proteins and/or nucleic acids, render the tissue highly transparent, and preserve the 3D cellular structures and fluorescence intensity by removing lipids (delipidation), hydroxyapatite (decalcification), pigments (decolorization), and embedding the tissue in an RI matching hydrophobic solvent or aqueous medium [61,81].

In the last decade, LSFM combined with optical tissue clearing has significantly impacted various fields such as neuroscience and developmental biology [64,77,82,83,84,85]. Tissue clearing methods were used to map the neuronal connections and neural activity in the mouse brain [72,73,86,87], generate whole-brain atlases with single-cell resolution [88,89], and complexly map, label, and regionally annotate the vascular topology of the normal mouse brain and in models of congenital deafness and ischemic stroke [90,91]. LSFM is also emerging as a powerful tool in cancer research for the 3D visualization and analysis of tumor biology, drug penetration, treatment response [92,93,94], and metastasis [92,95].

#### 3D Histopathology of Human Organs and Tumors

Modern pathology relies on thin sections (<10 μm-thick) of FFPE (Formalin-Fixed Paraffin-Embedded) tissue, which are subsequently stained with H&E (hematoxylin and eosin) or other diagnostic markers. However, solid tumors exhibit spatial heterogeneity and consist of various cell types and niches, vasculature, and distinct phenotypic features. Conventional histopathology is only capable of capturing a reduced aspect of the 3D spatial structures of intact tumors. Due to technological advances in LSFM and tissue clearing protocols, several studies have begun to bridge the gap and visualize whole human organs and tumors [96,97]. For example, DIPCO, a pipeline for reconstructing FFPE samples, was developed by applying deparaffinization, solvent-based iDISCO clearing, and LSFM to identify unique phenotypic heterogeneity in epithelial-mesenchymal transition and angiogenesis in cleared tumor biopsies from patients with solid tumors. This technique also allows re-embedding of cleared FFPE samples in paraffin for subsequent studies [98,99]. OTLS microscopes have also been used to enable rapid, non-destructive imaging of intact intra- and postoperative clinical specimens, as well as the volumetric assessment of optically cleared and labeled core-needle biopsies [67,100]. The CUBIC solution pipeline (hydrophilic reagents) has also been used to study human lung and lymph node tissue, which improved the detection sensitivity of small metastatic carcinoma nodules in lymph nodes, and is compatible with IHC and standard histology after rehydration [101]. Although a processing time of more than a week limits clinical applicability, the approach is nevertheless promising for revealing new aspects of tumor biology.

The CLARITY protocol (hydrogel-based) has also been used for 3D imaging of archived FFPE clinical samples from breast cancer tissues [102], for imaging different cell types such as neurons, astrocytes, and myelinated fibers in human brain tissue blocks [103], and for analyzing tissue blocks from the frontal lobe of an autistic patient [77]. An alternative protocol to CLARITY called SWITCH (System-Wide control of Interaction Time and kinetics of Chemicals) [104] was developed to enable multiplex imaging of human brain samples; another protocol called MASH (Multiscale Architectonic Staining of Human Cortex) was developed as a cytoarchitectonic labeling approach for optically cleared human cortex samples from formalin-fixed adult brain samples [105]. In normal tissues, the 3DISCO clearing method has been used to study gonadotropin-releasing hormone expressing neurons in intact human embryos and fetuses [106].

Recently, a new technique called pathoDISCO (hydrophobic reagents) has been developed and applied to breast cancer tissues. This technique uses selective autofluorescence enhancement, rapid chemical tissue clearing, and light-sheet microscopy to obtain virtual sections and 3D reconstructions of resected tumors with tissue size of ~30 × 20 × 5 mm^3^ processed in less than 48 h [107]. Selective autofluorescence enhancement is reversible; therefore, cleared tissue regions can be stained after with H&E or IHC and mapped back to the 3D reconstruction. A method called SHANEL was developed to clear human organs for light-sheet imaging. The entire process, including labeling and clearing, took ~4 months for an adult human brain and resulted in 56% shrinkage of the final brain volume [69]. This approach provides the basis for 3D histological assessment of the whole human brain in the near future.

Several challenges must be overcome before 3D imaging protocols can be successfully extended to the clinical setting. Time-efficient protocols for tissue clearing and multi-labeling techniques, as well as more cost-effective light-sheet microscopes and associated analysis software need to be developed. In contrast to the lipid-rich and homogeneous structures of the mouse brain, solid tumors often have an abundance of fibrous tissue and a heterogeneous composition, so visual assessment of tissue clearing between samples can be difficult. In general, the tissue-clearing methods (hydrophobic, hydrophilic, or hydrogel-based) can affect the morphology of some structures (expansion and shrinkage during the protocol), which can deform connected regions within the tissue. In addition, tissue shrinkage may interfere with accurate pathological assessment in routine clinical diagnostics. Therefore, clearing protocols must be optimized to achieve standardized and reproducible tissue processing methods for research and clinical applications.

Mouse xenograft tumor models can serve as preclinical models to optimize antibody penetration and concentration for protein markers of interest. In addition, the time required for tissue clearing and immunostaining needs to be reduced to make this technology useful for drug development and clinical research. There are also limitations in the availability of commercial image analysis software. More sophisticated and applicable software need to be developed to process large datasets along with machine learning algorithms to process feature extraction and meaningful 3D spatial relationships in the TME. Each of these described techniques represents a valuable approach for research purposes. However, they do not yet seem to be suitable in routine clinical histopathology due to long incubation times, the need for large volumes of antibodies along with issues related to antibody specificity, tissue perturbation and shrinkage due to different clearing methods.

Nevertheless, the rapid development of protocols for tissue clearing and the commercial availability of light-sheet microscope systems will one day outweigh the challenges in applying whole-tissue 3D imaging in the clinical setting. LSFM is likely to be highly informative for understanding tumor biology in patient samples.

### 3.2. Serial Two-Photon Tomography

Block-face imaging platforms such as STPT generate complete datasets that can be registered to standardized anatomical reference atlases, which are crucial for integrating anatomical data from multiple experiments. In this review, we focus on STPT, which takes advantage of two-photon excitation to optically section the standard paraformaldehyde fixed tissues [108]. Other block-face imaging modalities such as knife-edge scanning microscopy (KESM) [109] and micro-optical scanning tomography (MOST) [110] are also available. These technologies can produce complete 3D whole-brain imaging volumes using micrometer-scale sectioning and single-photon imaging of tissue embedded in resin.

STPT is an automated method developed for high-throughput ex vivo mosaic imaging of fluorescent-labeled brains and can employ three different PMTs [108]. Paraformaldehyde-fixed and agarose-embedded brains are alternately scanned with a high-speed two-photon microscope and sectioned with a vibratome. The STPT offers several advantages: (1) sliced brain sections can be indexed and automatically collected for subsequent histological or molecular analysis; (2) as the fixation and embedding steps are relatively simple, there is minimal adverse effect on brain morphology and fluorescence signal intensity, and (3) tissue distortion and sectioning artifacts are minimal due to subsurface scanning, allowing accurate imaging of serial images in 3D. Overall, this method is ideal for a variety of neuroanatomical projects involving normal and pathological conditions [108,111,112,113,114,115].

Automated imaging platforms, such as STPT, generate large amounts of data. The mouse brain can be imaged as a set of 280 serial coronal sections, with a *z*-spacing of 50 μm and an *x*/*y* resolution of 1 μm/voxel, within ~21 h [116]. STPT has an optical imaging capacity and a piezo objective scanner that can be used for *z*-stack imaging (2.5 μm spacing in *z*). STPT can provide a dataset ranging from hundreds of gigabytes for spatially down-sampled STPT brains [117,118] to the order of terabytes for large volume mouse brain datasets. Therefore, data analysis frameworks are being developed to visualize, map, and quantitatively analyze the mouse brain-derived imaging data. An STPT-based platform has been developed where fixed, gelatin-embedded, and cleared brains were imaged and reconstructed to generate a 3D image volume of the mouse brain to reveal long-distance axonal morphology [119]. New neuroinformatics tools were developed to create a standardized digital atlas of mouse brain datasets, which became known as the Allen Brain Atlas (ABA and Mouse Common Coordinate Framework (CCF)) [120]. STPT has been successfully used as part of the Allen Mouse Brain Connectivity Atlas pipeline [121]; therefore, studies using STPT can be accurately registered to the atlas to annotate anatomical references.

**Figure 3:**
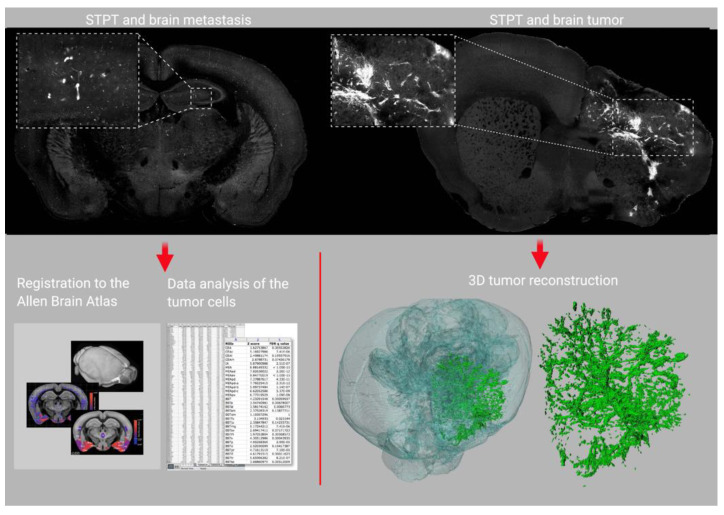
Fluorescent labeled tumor cells can be detected using serial two-photon tomography (STPT) (top panel), registered to a reference brain atlas (bottom left), and reconstructed in 3D (bottom right). Example STPT images are provided for preclinical brain metastasis (top left) and brain tumor (top right) models. The figure was created in BioRender.com (accessed on 12 March 2021) using our own preliminary data and exported under a paid subscription.

Although STPT is not yet widely used in the cancer field, this microscopy platform offers several advantages for the study of cancers, particularly brain tumors. First, STPT does not require optical clearing for imaging, and tissue morphology is preserved due to the light paraformaldehyde fixation. This aspect may be advantageous for the study of tumor–stroma interactions. In our previous work, we highlighted the usefulness of STPT in visualizing and quantifying the infiltration of implanted GFP-labeled mutant IDH1-expressing astrocytes in the mouse brain relative to reference anatomical structures annotated by the Allen Brain Atlas [122] (Figure 3).

Second, with the fourth generation of the STPT vibratome, tissue sections with a thickness of 20 to 25 μm are captured. This is very useful for the analysis of cell morphology. For instance, the indexed sections can be used for genomics, proteomics or stained for markers using traditional histology approaches after image acquisition. STPT can also be integrated into a tissue processing module, where thin sections from 25 μm upward are captured as they are cut from an agarose-embedded fixed whole organ. This module then transports these sections with a conveyer belt and automatically transfers and mounts them onto glass slides indexed back to their correct position in the overall image. This specific feature of STPT has the potential to reduce handling time of the sections to achieve a high throughput. Any subsequent data obtained from indexed sections can be easily overlaid on top of the 3D reconstructed imaging data to create a rich, multimodal dataset. Indexed sections also allow researchers to relabel and map tissue sections to answer follow-up questions. Since spatial integration of data acquired from multiple modalities is an important initiative in cancer, technologies that allow integration of 3D spatial reconstruction and molecular profiling of indexed tissue sections represent an ideal approach. Therefore, STPT sections can be processed for subsequent applications, providing a promising framework for application to patient samples.

Recently, several features of STPT have been improved. For example, to reduce the scan time and thus the acquisition time, a multiphoton, multi-foci scanning approach has been implemented. This approach splits the incoming laser beam into multiple spots scanned simultaneously across the sample and allows the full power of the laser system to be utilized by distributing the power across multiple spots avoiding saturation of the area excited by the laser. It also speeds up the scanning of an individual frame. For example, a 2-foci system can scan a single image twice as fast as a traditional one-foci system. Furthermore, the multi-foci systems do not suffer a reduction in the pixel residence and maintain the same signal-to-noise ratio as a single-foci galvo-based system. Reducing the sample scanning time, the replacement of intermediate optics allows a larger FOV to be scanned while maintaining the same image quality as the original system.

In the context of brain tumors, collagen is the major component of the TME and is involved in cancer fibrosis [123,124]. Second-harmonic generation (SHG) is a label-free, highly sensitive nonlinear optical technique that detects proteins, peptides, and adsorption of small-molecules [125]. STPT can visualize the collagen label-free and map the collagen network throughout the entire tumor. In SHG, two photons of equal energy are combined by a nonlinear material to emit a single photon with twice the energy. The collagen fiber distribution is detected at exactly half the wavelength of the excitation beam. For instance, excitation at 920 nm will produce an SHG signal at 460 nm, and the SHG signal can be identified with a bandpass filter at 460 ± 10 nm in front of the PMT to help filter any fluorescence signals. In addition, third-harmonic generation (THG) is another nonlinear excitation method. THG signals arise in regions where the RI changes significantly, e.g., along an interface. THG is expected to be detected at exactly one-third of the excitation wavelength. In the context of the brain, it is a useful method to perform label-free imaging of myelin sheaths.

STPT has some drawbacks; for example, volumetric data acquisition rates for block-face imaging methods are slower compared to light-sheet imaging and multi-color imaging, and antibody labeling have not yet been adapted to STPT. However, tissue clearing in conjunction with antibody labeling protocols will likely be adapted for STPT in the near future. Since the tissue is only lightly fixed, the structure and volume of the sample are preserved, which offers advantages for downstream applications that apply molecular or histological techniques. Furthermore, given the analytical neuroscience pipelines already available, STPT can be readily used to study brain tumors.

## 4. Conclusions

A comprehensive understanding of brain tumors needs to consider the spatial structures of tumors, their TME, and their localization within the host environment. The rodent brain has been extensively studied to understand its neural circuitry, normal function, and pathology using 3D technologies, and researchers are in the process of studying the 3D structures of extracranial solid tumors from patients using ex vivo 3D imaging approaches. As such, the field of neuro-oncology needs to take advantage of the many advances, as these new methods may have important implications for personalized medicine and can improve our understanding of this often fatal disease. As imaging technologies and analysis pipelines improve, STPT and LSFM are positioned to provide important fundamental insights into the pathology of brain tumors.

## Figures and Tables

**Table 1:** Summary of commonly used fluorescent probes for two-photon intravital microscopy.

Labeling Target	Fluorescent Probe
Tumor cells	CMTMR
	CFSE (green)
Vasculature	Dextrans
	Quantum dots Angiosense probes Fluorescent lectin
Neural networks	AM esters of Ca^2+^ indicators
Macrophages/microglia/monocytes	Iron oxide nanoparticles
Cathepsins	Prosense CatB
Metalloproteinases	MMPsense
Integrin receptor	IntegriSense
